# Multiple giant scalp metastases of a follicular thyroid carcinoma

**DOI:** 10.1186/1477-7819-6-82

**Published:** 2008-08-11

**Authors:** Kenko Cupisti, Uwe Ramp, Andreas Raffel, Markus Krausch, Alexander Rehders, Wolfram T Knoefel

**Affiliations:** 1 Department of General, Visceral and Pediatric Surgery, University Hospital, Heinrich-Heine-University of Düsseldorf, Germany; 2 Institute of Pathology, University Hospital, Heinrich-Heine-University of Düsseldorf, Germany

## Abstract

**Background:**

The occurrence of skin metastases are rare events in the course of a follicular thyroid carcinoma (FTC) and usually indicate advanced tumor stages. The scalp is the most affected area of these metastases.

**Case presentation:**

We present a case of a 76 year old Woman with multiple giant scalp metastases of a follicular carcinoma. These metastases had been resected and wounds had been closed with mesh graft. The 14-months follow up is presented.

**Conclusion:**

We demonstrate another case with multicentric form. Because of its location and size a primary wound closure was not possible. A healing could be reached using vacuum therapy and mesh graft transplantation.

## Background

The occurrence of skin metastases are rare events in the course of a follicular thyroid carcinoma (FTC) and usually indicate advanced tumor stages. The scalp is the most affected area of these metastases [1-6]. Operations are mostly performed with palliative intention. We present a case with extensive and symptomatic scalp metastases in a female patient. The tumors were resected under general anaesthesia. Mesh graft was successfully used to cover the skin defects.

## Case presentation

**Figure 1:**
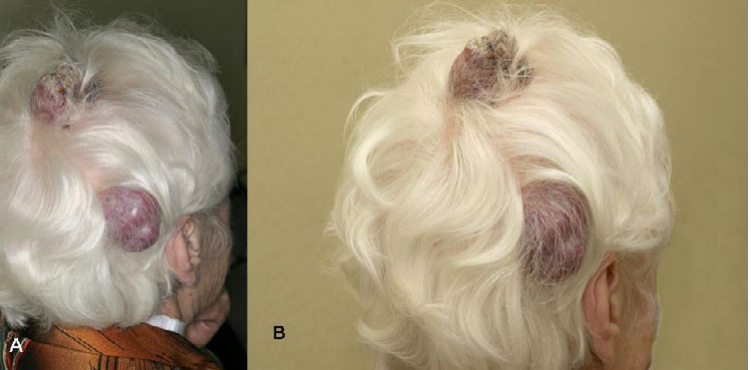
**a) and b) Two scalp tumors at admission of the patient, lateral view**.

**Figure 2:**
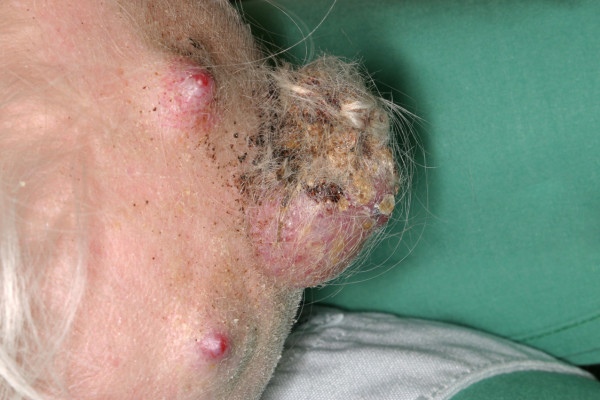
**Giant parietal scalp tumor and to additional smaller tumors, intraoperative view**.

**Figure 3:**
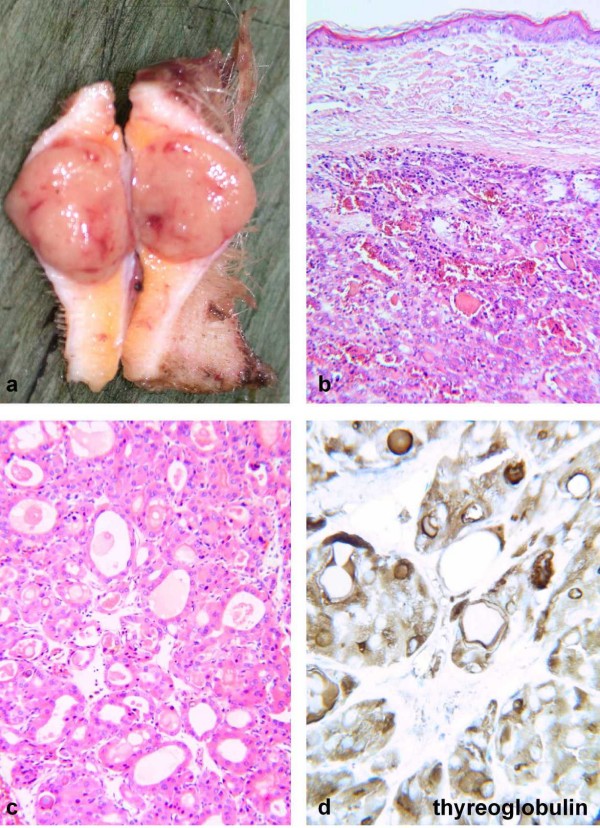
Operative specimen, Haematoxylin-Eosin and Thyreoglobulin staining.

**Figure 4:**
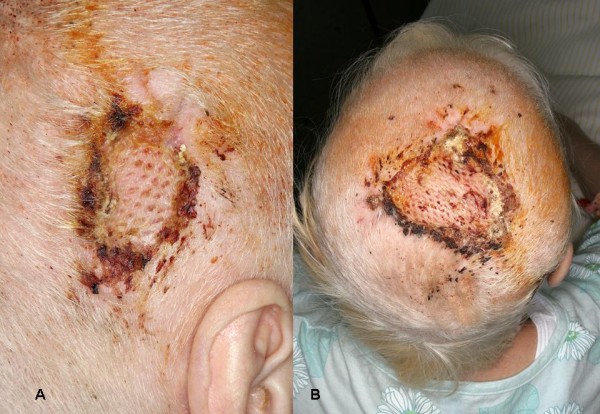
**Mesh Graft transplants**. a) Retroauricular mesh graft transplant; b) Parietal mesh graft transplant.

A 76-year old female patient had the initial diagnosis of FTC 18 years ago. She had total thyroidectomy with bilateral neck dissection and multiple reoperations for recurrent tumor. Because of an irresectable local recurrence with tracheal infiltration a tracheotomy was performed two years ago. Five sets of internal radiation therapy, had been performed one year ago with a cumulative activity of 55.400 MBq^131^I. She was admitted to our hospital because of four intensively vascularized scalp tumors, two of them of hen's egg size (Fig. 1a, b, and 2) which showed recurrent episodes of contact bleeding during hair dressing. Computed tomography revealed multiple pulmonary, hepatic and bone metastases. Thyreoglobulin level was highly elevated (6750 ng/ml) Nevertheless the patient was in a good general condition. We performed a resection of the scalp tumors under general anesthesia. Histopathology confirmed cutaneous metastases of FTC (Fig. 3). The places of resection were primary left for granulation. After achievement of a clean granulation area using vacuum therapy (*V.A.C.^®^, KCI International, Amsterdam, The Netherlands*) we performed a mesh graft skin transplant (Fig. 4a, b).

**Figure 5:**
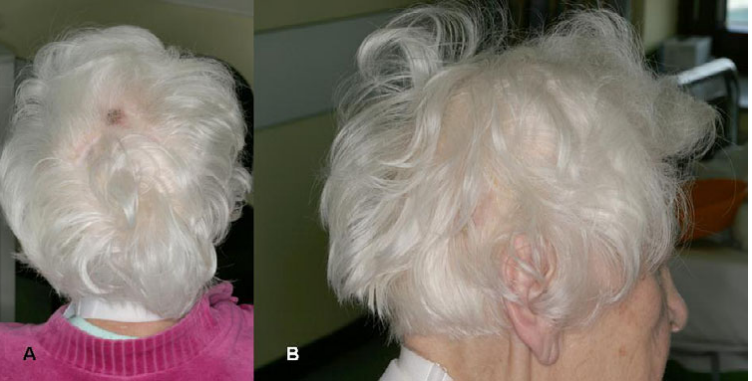
**14 months follow up**. a) view from on high; b) lateral view.

A follow up examination fourteen months later showed a very good cosmetic result with nearly complete healing of the mesh graft transplant (Fig. 5a, b). Because the local neck tumor had continued to grow the patient was now convinced to accept external radiation therapy and was admitted to our department of radiation oncology.

## Discussion

Follicular thyroid carcinomas (FTC) often spread to bones and lung [7]. The occurrence of cutaneous metastases is a rare event. Many different locations have been decribed as abdomen, back and front thigh [8], but predominantely the skin of head and neck is affected [1-6]. In a review of the literature Quinn and coworkers [1] found scalp metastases in 9 of 14 patients with cutaneous metastases of FTC. In a study of Erickson and coworkers [9] none of 5 FTC metastatic to the skin showed BRAF(V600E) mutation (T1799A).

## Conclusion

We demonstrate another case with multicentric form. Because of its location and size a primary wound closure was not possible. A healing could be reached using vacuum therapy and mesh graft transplantation. The palliative long term cosmetic and functional result was excellent.

## Competing interests

The authors declare that they have no competing interests.

## Authors' contributions

KC had idea to publish the case report and drafted the manuscript, UR was our pathologist and performed the immunohistochemistry, AR and MK helped to to search and analyse thoroughly the literature, AR and WTK performed the initial operation, mesh graft transplantation and follow up examination of the patient. They also initiated the temporary vaccum therapy. All authors read and approved the final manuscript.

## Consent

Written informed consent was obtained from the patient for publication of this case report and any accompanying images. A copy of the written consent is available for review by the Editor-in-Chief of this journal.
